# Network-Based Approach and IVI Methodologies, a Combined Data Investigation Identified Probable Key Genes in Cardiovascular Disease and Chronic Kidney Disease

**DOI:** 10.3389/fcvm.2021.755321

**Published:** 2022-01-05

**Authors:** Mohd Murshad Ahmed, Safia Tazyeen, Shafiul Haque, Ahmad Alsulimani, Rafat Ali, Mohd Sajad, Aftab Alam, Shahnawaz Ali, Hala Abubaker Bagabir, Rania Abubaker Bagabir, Romana Ishrat

**Affiliations:** ^1^Centre for Interdisciplinary Research in Basic Sciences, Jamia Millia Islamia, New Delhi, India; ^2^Research and Scientific Unit, College of Nursing and Allied Health Science, Jazan University, Jazan, Saudi Arabia; ^3^Department of Medical Laboratory Technology, College of Applied Medical Sciences, Jazan University, Jazan, Saudi Arbia; ^4^Department of Bioscience, Jamia Millia Islamia, New Delhi, India; ^5^Centre for Stem Cell & Regenerative Medicine, KING' College London, Guy's Hospital, London, United Kingdom; ^6^Department of Medical Physiology, Faculty of Medicine, King Abdulaziz University, Rabigh, Saudi Arabia; ^7^Department of Hematology and Immunology, College of Medicine, Umm-Al-Qura University, Mecca, Saudi Arabia

**Keywords:** CVD, CKD, PPIN network, IVI, hubness score, spreading score

## Abstract

In fact, the risk of dying from CVD is significant when compared to the risk of developing end-stage renal disease (ESRD). Moreover, patients with severe CKD are often excluded from randomized controlled trials, making evidence-based therapy of comorbidities like CVD complicated. Thus, the goal of this study was to use an integrated bioinformatics approach to not only uncover Differentially Expressed Genes (DEGs), their associated functions, and pathways but also give a glimpse of how these two conditions are related at the molecular level. We started with GEO2R/R program (version 3.6.3, 64 bit) to get DEGs by comparing gene expression microarray data from CVD and CKD. Thereafter, the online STRING version 11.1 program was used to look for any correlations between all these common and/or overlapping DEGs, and the results were visualized using Cytoscape (version 3.8.0). Further, we used MCODE, a cytoscape plugin, and identified a total of 15 modules/clusters of the primary network. Interestingly, 10 of these modules contained our genes of interest (key genes). Out of these 10 modules that consist of 19 key genes (11 downregulated and 8 up-regulated), Module 1 (RPL13, RPLP0, RPS24, and RPS2) and module 5 (MYC, COX7B, and SOCS3) had the highest number of these genes. Then we used ClueGO to add a layer of GO terms with pathways to get a functionally ordered network. Finally, to identify the most influential nodes, we employed a novel technique called Integrated Value of Influence (IVI) by combining the network's most critical topological attributes. This method suggests that the nodes with many connections (calculated by hubness score) and high spreading potential (the spreader nodes are intended to have the most impact on the information flow in the network) are the most influential or essential nodes in a network. Thus, based on IVI values, hubness score, and spreading score, top 20 nodes were extracted, in which RPS27A non-seed gene and RPS2, a seed gene, came out to be the important node in the network.

## Introduction

The risk of getting cardiovascular disease (CVD) in patients with chronic kidney disease (CKD) is more than without CKD as discussed by Jankowski et al. (1). Chronic kidney disease (CKD) is a systemic condition that affects almost 10% of the population. The prevalence of CKD has increased in recent decades due to aging which affects about one out of every 10 people (2). Multiple studies have confirmed that individuals with renal disease undergo rapid aging, which accelerates the onset of pathologies, such as CVD, which is strongly correlated with older age. Furthermore, patients with CKD are more prone to CVD and even death due to the progression of end-stage renal disease (ESRD) (3). CVD, along with chronic renal disease, remains the leading cause of morbidity and mortality in individuals, particularly in those involving a systemic inflammatory process, such as atherosclerosis (4). CKD is the 14th leading cause of mortality, with the death rate anticipated to increase to 14 per 100,000 people by 2030 (5). Despite the rising pervasiveness of CKD and its frequent combination with CVD, patients with severe CKD (eGFR 30 ml/min per 1.73 m2) were commonly omitted from key randomized controlled studies (6). Traditional CVD risk factors, such as hypertension, advanced age, diabetes, male sex, dyslipidemia, and smoking, are also prevalent in CKD. The number of people with end-stage renal disease (ESRD) is expected to rise by 50% in the next 20 years (7). In the current era, CKD is a severe health and economic burden. CKD-related mortality has increased by 82.3% in the last two decades. In addition, it has risen to third place among the world's top 25 major causes of death (8). Now, the molecular description of CKD onset and progression is lacking. Based on these findings, the researchers have described CKD as a worldwide epidemic (9). The present method for prioritizing disease-related genes is based on the “guilt-by-association” assumption, which states that physically and functionally related genes have similar phenotypic effects and are likely to be involved in the same biological pathways (10).

Network theory is a useful tool for deciphering the topological features and dynamics of complex systems and their functional modules. Many extant networks can be classified as scale-free, small world, random, or hierarchical networks. The hierarchical form of the network includes modules and sparsely dispersed hubs to manage the network which is of particular interest to biologists (11). Moreover, the identification of essential and critical protein(s) is crucial for understanding progression of the disease. Thus, the current paradigm for investigating CVD and CKD focuses on combining protein interactions, functions, and disease networks to identify important regulators of CVD and CKD among DEGs. Also, it is substantial to study network's topological characteristics, so that the essential key regulators, their function, and regulating mechanism could be forecasted (12). The topology of a network and its many centrality measures (metrics reflecting the impact of each node within a network) can be examined and evaluated to explore fundamental biological meanings and prominent regulatory molecules (13). A network could best be studied in relation to the spreader nodes as these are projected to have the biggest impact on the process of distribution of information throughout the network because they have strong connections with other nodes. So these influential nodes can commonly be identified by calculating the characteristics like hubness and spreading potential (14). Since the fundamental characteristics of a network and its centrality metrics are universal, it can be applied to any network domain, including biological networks (15). These network centralities can be used to further narrow down and validate the extracted influential nodes. In our present study, we have used the seed genes, gene ontology, and pathways in an integrating manner to give a glimpse of molecular relation between CVD and CKD that may further facilitate the identification and validation of novel biomarkers.

## Methodology

### Acquisition of mRNA Expression Data

The mRNA expression profile of CVD and CKD were downloaded from the publicly available databases of gene expression microarrays, stored in the repository bank Gene Expression Omnibus (GEO) NCBI [www.ncbi.nlm.nih.gov/geo/; (16)]. These were the mRNAs expression profiles series of CVD (GSE26887, GSE42955, GSE67492, GSE71226, GSE141512, GSE48060) and CKD (GSE15072, GSE23609, GSE43484, GSE62792, GSE66494) consisting of normal and treated samples. The selection of the datasets using inclusion and exclusion criteria were made using the following keywords in the NCBI : CVD, cardiac failure, cardiac arrest, chronic heart failure (CHF), etc., whereas, CKD, AKI, chronic kidney disease, renal failure AND human [Organism] (17). The details of all GSE series are given in Table 1. Patient samples from various sources were not differentiated during the data integration procedure to show a common and/or overlapped gene signature. The expression microarray is a method for studying gene expression on a genome-wide scale that is widely utilized. Batch effects can be decreased through proper experimental design, but they cannot be eradicated until the entire study is conducted in one batch. Before analyzing microarray data, a few algorithms are now available to correct for batch effects. We employed the Empirical Bayes method built-in function in LIMMA, in combination with the fit2 function. Meta-analyses based on microarray data integration require effective *in silico* methods. We may now use *in silico* tools to efficiently merge numerous microarray datasets while ignoring differing demographics, experimental designs, and specimen sources with the advancements of ever-growing theories and bioinformatics tools (18).

**Table 1:** Samples from the GSE series and their DEGs.

**SERIES**	**Total sample**	**Normal**	**Disease**	**Up regulated**	**Down regulated**	**Fold change**	**Illness**	**Country**	**Year**	**Platform**	**Authors**	**Source**
GSE48060	52	21	31	15	14	0.5	CVD	USA	2014	GPL-570	Xing Li	Peripheral blood
GSE67492	6	2	4	73	16	0.5	CVD	USA	2015	GPL-6244	James West	Right ventricular wall
GSE26887	24	5	19	58	95	0.5	CVD	Italy	2012	GPL-6244	Fabio Martelli	Left ventricular wall
GSE71226	6	3	3	97	82	1.5	CVD	China	2015	GPL-570	Bofan Meng	Peripheral blood
GSE42955	29	5	24	125	236	0.5	CVD	Spain	2013	GPL-6244	M. M. Molina	Heart
GSE141512	12	6	6	69	85	0.5	CVD	Russia	2019	GPL-17586	German Osmak	Blood
GSE66494	61	8	53	102	325	0.5	CKD	Japan	2015	GPL-6480	Satohiro Masada	Kidney
GSE43484	6	3	3	134	136	0.5	CKD	Sweden	2013	GPL-571	Elham Dadfar	Uremic monocyte
GSE15072	29	8	21	51	38	2	CKD	Italy	2009	GPL-96	Palo Pontrelli	PBMC
GSE62792	18	6	12	352	262	0.5	CKD	Sri Lanka	2018	GPL-10558	D. N. Magana	Blood
GSE23609	24	7	17	219	189	0.5	CKD	USA	2010	GPL-6454	Persis P. Wadia	Serum

*The entire sample available in the GSE series is shown in column 2, and the fold change values are shown in column 7. In each GSE series, the total up and down regulated genes were recorded in 5 and 6 columns, respectively. For the GSE series, the platform GPL in column 11 indicates the number of probes and the type of data (Affymetrix data and oligo data)*.

### Identification of CVD and CKD Associated DEGs

To further analyze the obtained data samples series, the GEO2R tool (http://www.ncbi.nlm.nih.gov/geo/geo2r/) was used. GEO2R is a web-based analytical tool with a built-in R program and GEO query for Linear Models for Microarray Data (Limma). The default settings were utilized to prepare the datasets (19). Differentially Expressed mRNAs were extracted applying criteria *p* < 0.05 and log fold-change |0.5–2| as the threshold values. Up and downregulated genes from meta-analysis were used as genes of interest in PPIN network. As a result, the Benjamini-Hochberg (BH) correction method was utilized to adjust the significant value of *p* obtained by the original hypothesis test during differential expression analysis. Finally, for DEGs screening, log Fold change was employed as the key index. *Log fold change* |0.5–2| was employed as a DEG screening condition in this study. The criteria [i.e., value of *p* < 0.05 and fold change 0.5–2] were selected in order to expand the total number of DEGs between healthy control and diseases samples. Since very few genes were differentially expressed with respect to this threshold, therefore it made the threshold more stringent and would lead to nearly no or very few DEGs. If the fold change is altered, the resulting DEGs will be changed as well, and vice versa. There is no standard value for fold change when it comes to selecting DEGs (20).

### Network Construction of Protein-Protein Interactions (PPIN)

The network was built using the String online database and imported into Cytoscape v.3.80. It supports bipartite graph visualization of gene-gene linking/interaction/regulation (gene-disease associations) and also provides gene-centric views of the network data (21). The Probe Ids were mapped to their corresponding gene symbols to build the native network from DEGs. The gene regulatory network of CVD and CKD was built using the simple premise of one gene and one protein. A variety of features can be used to construct and filter the network. The network combines data from curated databases with knowledge gleaned from the literature (22). Because the networks might be based on a certain gene or condition, the PPIN networks are visualized using the Cytoscape program (23). To discover the significant modules and top-ranked genes in the PPIN network, the Cytoscape plugin Molecular Complex Detection (MCODE; version 2.0.0) and the igraph with Influential packages in R (version 3.6.3) were used. The network's topological properties were estimated using Network analyzer, a Cytoscape version 3.8.0. plug-in, while the eigenvalues were generated using CytoNCA, another Cytoscape plug-in for topological properties calculation. This could also be useful for double-checking Network Analyzer data (24).

### Module/Cluster/Subnetwork Finding: Molecular COmplex DEtection Method

The MCODE method (A cytoscape plugin) was used to detect the modules/clusters/communities (Strongly interconnected regions) in the native network. In a PPI network, protein complexes and pathways are prevalent groupings (clusters) (25). To separate the dense regions according to provided parameters, the approach uses vertex weighting by local neighborhood density and outward traversal from a locally dense seed protein. The algorithm has the advantage of having a directed mode, which permits fine-tuning of clusters of interest without considering the remainder of the network and investigation of cluster interconnectivity, which is important in protein networks (26). The modules from the native network along with sub-modules from modules at each level of organization were identified until only motifs remained (i.e., 3 nodes with 3 edges). Vertex weighting, complex prediction, and possible post-processing to filter or add proteins in the generated complexes are the three stages of the MCODE algorithm (27). Intuitively, a network of interacting molecules can be represented as a graph with vertices representing molecules and edges representing chemical interactions. If the temporal pathway or cell signaling information is known, a directed graph with arcs reflecting chemical activity or information flow can be created. Otherwise, an undirected graph is employed. Graph theoretic methods can be used to aid in the analysis and solution of biological problems using this graph model of a biological system (28).

### Gene Ontology, Pathway Analysis, and HE

Gene ontology words are a standardized set of terms separated into three categories: Molecular function, Biological Processes, and Protein Class (29). Thus, DEGs were collected and sent to ClueGO (a Cytoscape plug-in that facilitates biological interpretation and visualizes functionally grouped terms in the form of networks, tables, and charts) to enrich the given set of DEGs to possible GO terms for exploratory research. We used ClueGO to assess the function of hub genes and define the GO term. ClueGO employs kappa statistics to link the terms in the network. It creates a dynamical network structure by considering the gene lists of interest at the start. It also constructs a functionally ordered GO/pathway term network by combining GO terms and KEGG/BioCarta pathways (30). A number of adjustable limitation criteria allow for visualizations of various levels of specificity. ClueGO can also compare gene groups and highlight their functional differences. ClueGO also makes use of Cytoscape's flexible visualization framework and works in tandem with the GOlorize plug-in. Gene lists can be imported directly into ClueGO or produced interactively from gene network graphs shown in Cytoscape. ClueGO comes with a few gene identities and species by default, and it is simple to add more via a plug-in. ClueGO is an open-source Java application that uses different ontologies to extract non-redundant biological information for huge clusters of genes (31).

For gene annotation and gene list enrichment analysis, Metascapes leverages a variety of databases and technologies. Metascape uses lists of gene identifiers to extract rich annotations, find statistically enriched pathways, and build PPIN networks. Pathway or process enrichment analysis uses the standard accumulative hypergeometric statistical test to identify ontology words with significant input genes given a gene list (32). We give more arguably better ontology concepts, including ones from Broad's MSigDB, than other GO-based enrichment analysis tools. Modules were also subjected to MCODE to identify sub-modules and sub-sub-modules. All modules, sub-modules, and sub-sub-modules with clustering coefficients less than or equal to unity were examined. We discovered modules and sub-modules that were related to independent functions and followed the modularity laws, and the fact that their activities are non-linear (33). As a result, we needed to quantify their role as important genes at the systems biology level, which we did by utilizing HE analysis of biological networks (34).

### Identification of the Most Influential Nodes Using IVI Methods

The IVI is a new method for detecting influential nodes. Hubness and spreading values combine to form the IVI algorithm. IVI captures all the network's topological dimensions. In domains ranging from transportation to biological systems, identifying critical individuals within a network is a persistent challenge. Identifying the most powerful nodes with the ability to have the most influence on the PPIN network (35), degree centrality (DC) and ClusterRank (CR), neighborhood connectivity (NC), local H index, betweenness centrality (BC), and collective influence (CI), respectively, and to synergize their effect for the unbiased identification of prominent nodes in the network (36). Influential is an R package that primarily focuses on identifying the most influential nodes in a network, as well as categorizing and rating top candidate characteristics (37).

## Results

**Figure 1:**
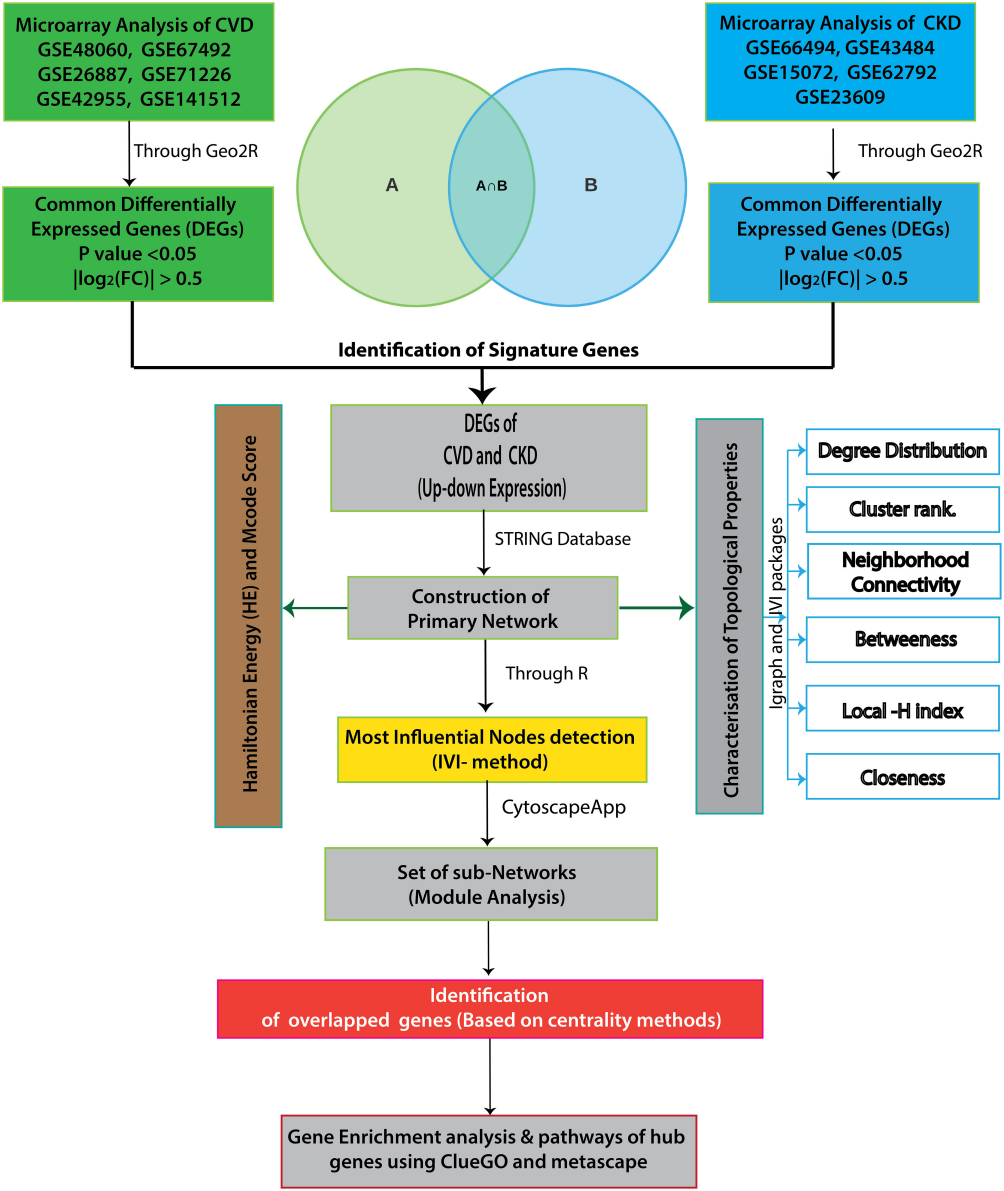
The workflow of our integrative network-based approach is depicted in this diagram.

Figure 1 depicts the workflow of the entire integrative network-based approach used in this study. Adobe Illustrator CS6 was used to create the flowchart.

### Selection of DEGs

The GSE series of CVD denoted by X and the GSE series of CKD was denoted by Y. There are 6 GSE series of X (X_1_, X_2_, X_3_, X_4_, X_5_, and X_6_) and 5 GSE series of Y (Y_1_, …… Y_5_).


(1) $$\begin{array}{ccc} Xu & = & \left(X_{1}u_{1},\;X_{2}u_{2},\ldots .X_{6}u_{6}\right) \end{array}$$

where u stands for upregulation.


(2) $$\begin{array}{ccc} Xd & = & \left(X_{1}\;d_{1},\;X_{2}d_{2}....\;X_{6}d_{6}\right) \end{array}$$

d stands for downregulation.


(3) $$\begin{array}{ccc} Yu & = & \left(Y_{1}\;u_{1},\ldots .Y_{5}u_{5}\right) \end{array}$$

(4) $$\begin{array}{ccc} Yd & = & \left(Y_{1}d_{1},\ldots .Y_{5}d_{5}\right) \end{array}$$

To find DEGs, we combine equations, i.e., (1) with (3) and (2) with (4), as follows:


(5) $$\begin{array}{ccc} Xu\cap Yu\; & = & \left\{Xu\right\}\;\cap \;\left\{Yu\right\} \end{array}$$

(6) $$\begin{array}{ccc} Xd\cap Yd\; & = & \left\{Xd\right\}\;\cap \;\left\{Yd\right\} \end{array}$$

**Figure 2:**
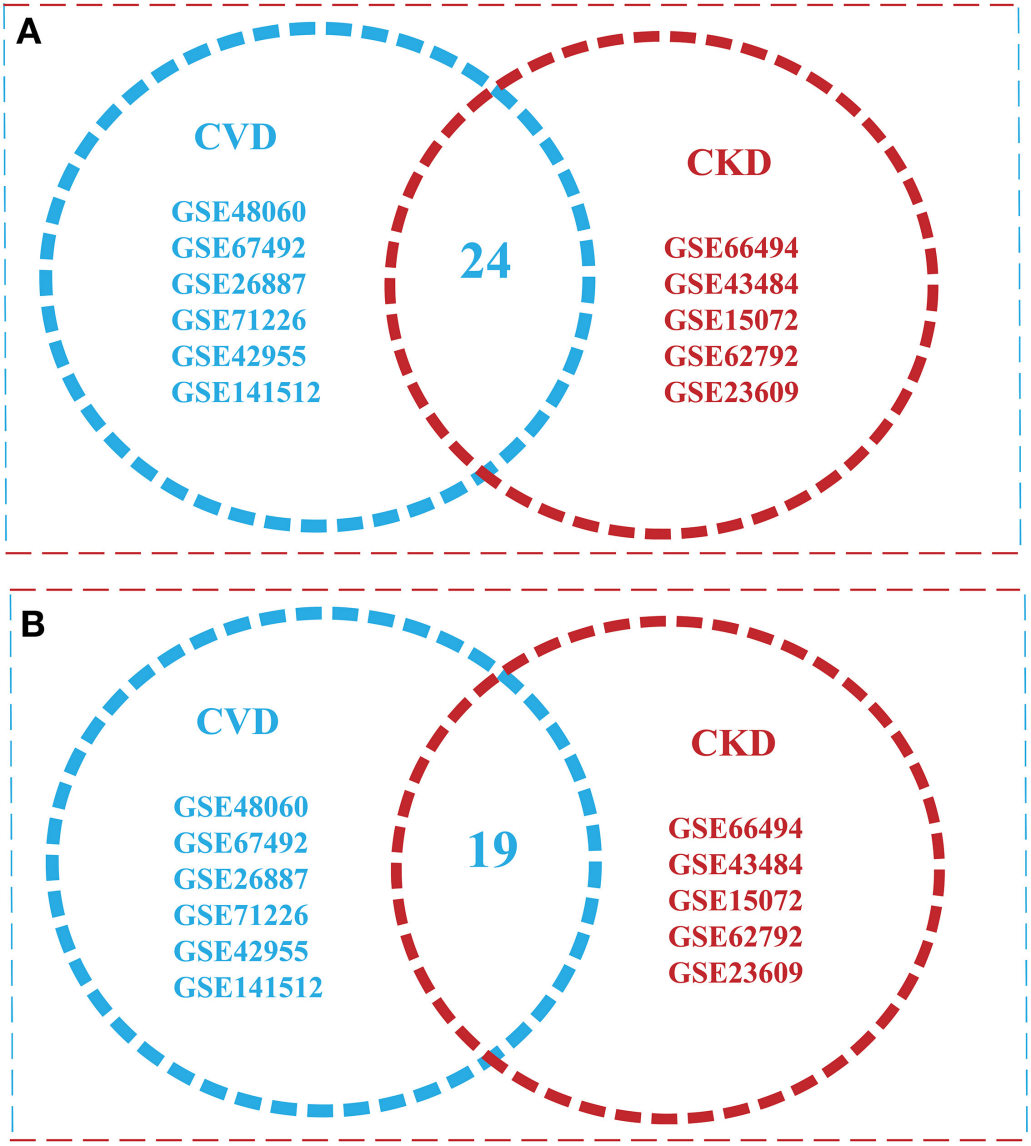
Venn diagram illustrating overlap of the two conditions with each other [cardiovascular disease (CVD) vs. chronic kidney disease (CKD)]. **(A)** Overlapped Upregulated genes in 11 GSE Series of CVD and CKD **(B)** a total of 24 downregulated genes were found in both conditions.

The intersection of (5) and (6) gives the Overlapped genes. Genes showing a value of *p* ≤ 0.05 and *log fold change* |0.5–2.0| were considered statistically significant and differentially expressed. The total upregulated genes in all 11 GSE series of CVD and CKD was *Xu*∩*Yu* = {1295}, whereas total downregulated genes are *Xd*∩*Yd* = {1478}. Total up and downregulated genes in CKD and CVD, separately, are 437 and 528, and 858 and 950 while the total upregulated genes in all 11 GSE series of CVD and CKD are 1,295, and downregulated genes are 1478 (Table 2). After comparison of CVD vs. CKD, we finally got 43 overlapped genes, including 24 upregulated genes in CVD and CKD, and 19 genes downregulated in both diseases (Figure 2).

**Table 2:** The up and downregulated genes are listed in a table with a count number column.

**Sr. No**.	**Common genes in CVD and CKD**	**# Count**	**Gene symbol**
1	UP regulation	19	NPR3, NFE2L1, TNFSF10, HMGB2, GABPB2, KDM5D, RSRP1, GPCPD1, ZNF83, THY1, COX7B, NPPB, PDZK1IP1, BCL6, IRAK3, GRN, SOCS3, CASP5, RPS24
2	Down regulation	24	BCL3, ZRANB2, NR1D2, C7, LYN, ANXA3, PER3, PTP4A3, RPLP0, HSPB1, ACTG1, RPL13, HCAR3, FCGR3A, MAP2K3, MYC, CIRBP, AHSA2, ATP1A1, NPIPB3, PNISR, RPS2, ENO1, CNN1

*Up-regulated genes have red lettering, whereas downregulated genes have green text*.

### Gene Network Construction

Based on mRNA profiling and protein networks, to generate subnetwork biomarkers (interconnected genes whose aggregate expression levels are predictive of disease state), networks provide a rich source of biomarkers for illness classification. To construct a CVD and CKD interaction network and infer gene-disease correlations using network features (38), we started with a list of seed/key genes (43 DEGs) that are known to be associated to the disease, and each gene is represented by a single node in the interaction network. Next, we built a disease-specific gene-interaction network where the nodes are the genes and the edges are their connections (39) using selection of top ranked genes in the network via IVI -Value, Spreading score, Hubness score, ClusterRank, Collective influence, h-index, Local h-index degree centrality (D_C_), betweenness centrality (B_C_), and Neighborhood Connectivity (N_K_) centrality metrics.

**Figure 3:**
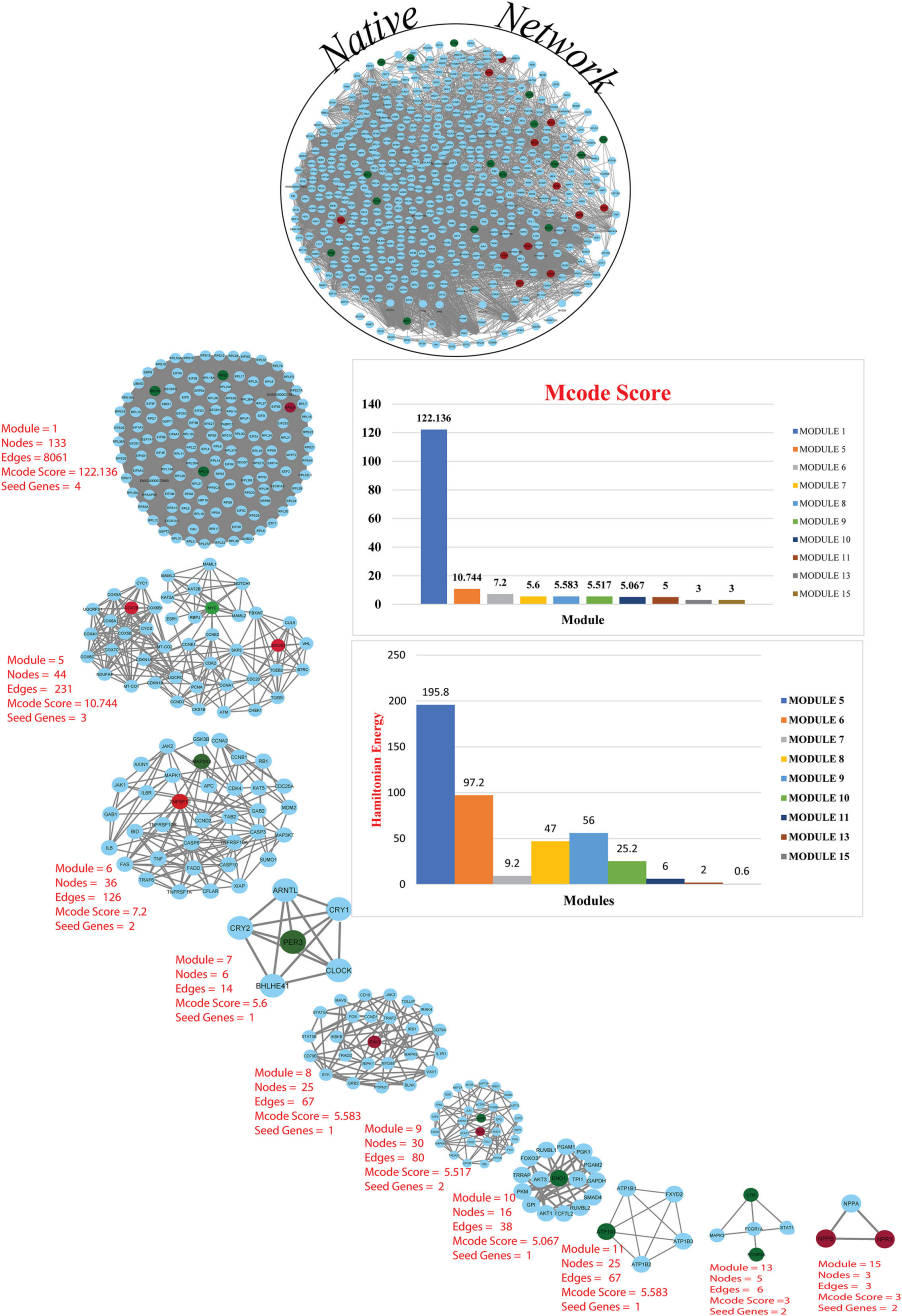
Cardiovascular disease and CKD parental network (merge network) with 587 nodes and 13,887 edges, red and green colors for seed genes and blue for interaction partners (non-seed genes). Using the MCODE cytoscape plugin, the native network is broken down into subnetworks up to the motif level. Each module subnetwork from native network represents with MCODE score and seed genes (up-regulated red and downregulated green). The hierarchy of energy is depicted on the right side of the illustration by a Hamiltonian energy (HE) bar graph. As the number of nodes in the modules decreases, so does the amount of energy used. The HE was calculated for nodes at each level of all conceivable modules in the network. The HE of the CVD and CKD networks is given as a function of network levels. We determined that energy distribution is greatest in the core network and diminishes as the level of (modules) organization increases. Because HE is dependent on nodes and edges for a fixed resolution parameter value (gamma = 0.8), the drop in HE shows the dominance of interacting edges over the network size, indicating rapid information processing. The HE of a complex network is a measure of overall energy in the system, and its value fluctuates as the network structure changes.

A network representation and analysis is a powerful tool for studying the complicated behavior of biological systems' physiology and pathology (40). Through cluster/module analysis, it was studied that the CVD and CKD network shows hierarchical scale free nature. The 19 key genes and their presence in various modules are traced following MCODE community finding algorithm. There are 15 modules (highly connected nodes) extracted from the native network. Ten out of 15 modules were found to be having our seed genes (Figure 3). These genes will be utilized to build a gene network and to investigate their biological importance.

### Gene Enrichment Analysis and Pathways

**Figure 4:**
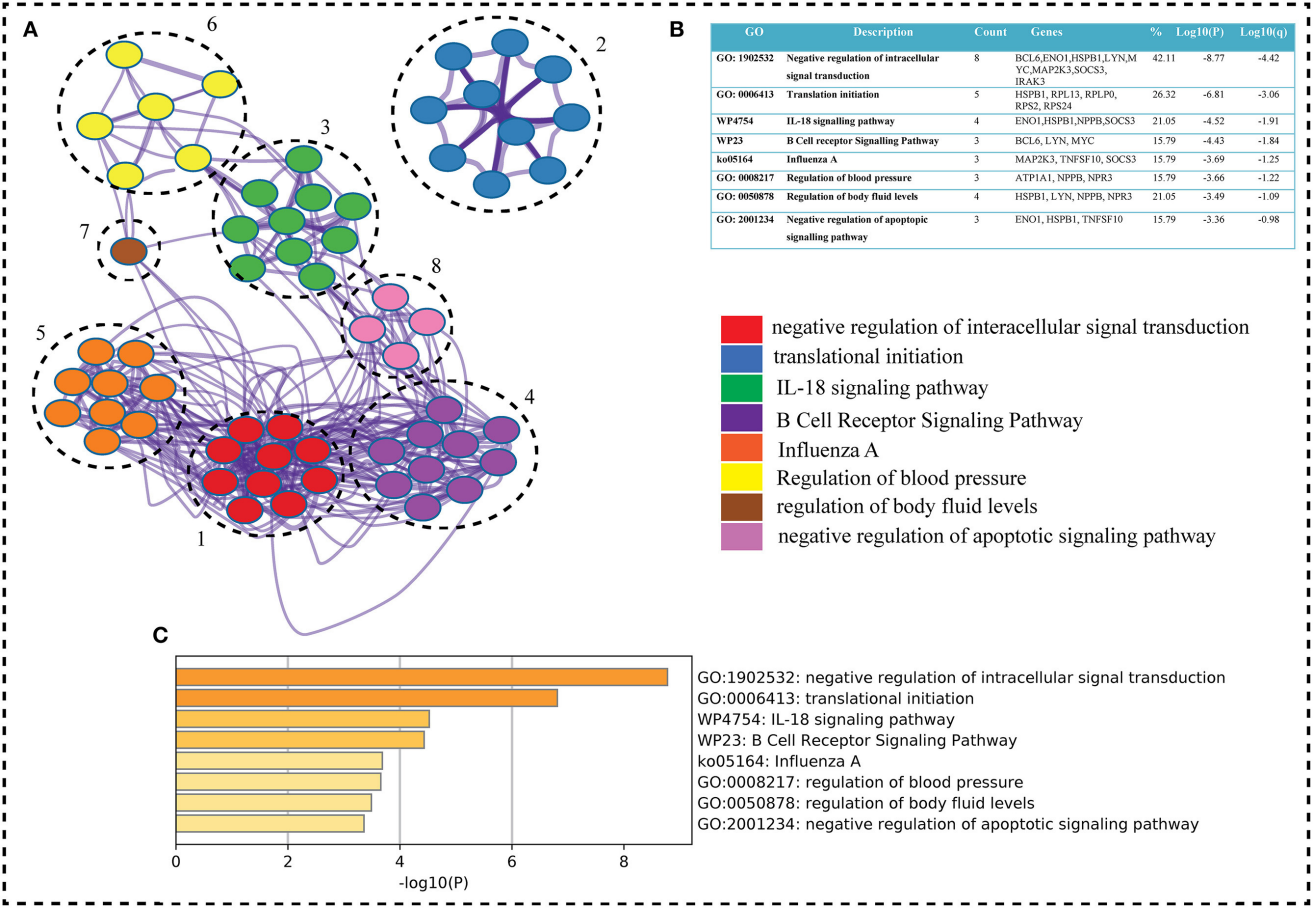
**(A)** Metascape, a web-based application, was used to enrich the gene ontology of our interest genes (seed genes). Metascape created an 8-cluster network for the go term (Biological Process), with color according on gene proportion. Each cluster has a large number of genes, including seed genes. Each cluster's node has a different GO term, but the main term is taken into account because of the seed genes (8 genes in first term and seed genes count decreases from top to bottom term whereas Log *P*-value increases). The GO keywords, gene counts, gene symbols, and lop *p*-value are shown in **(B)** table shows GO terms, genes count, genes symbols, and lop *p*-value. The term having seed genes count and log *p*-value higher were on top. In the GO term table w and k represents wiki and keg pathways, respectively. **(C)** Bar graph on the basis of *p*-value shows the GO term. As *P*-values decreases the go term goes top to bottom.

The key genes are submitted to ClueGO a cytoscape plugin to find the Gene Ontology term. The default setting of ClueGO are network specificity (medium), organism (human), visuals styles (group), evidence of experiments, all experimental (EXP, IDA, IPI, IMP, IGI, and IEP), and value of *p* < 0.05 (41). ClueGO constructs a functionally ordered GO/pathway term network by combining Gene Ontology (GO) terms and KEGG/BioCarta pathways. It can evaluate and compare numerous lists of genes and visualize functionally grouped terms in a comprehensive way. ClueGO can be used in conjunction with the GOlorize plug-in to offer an intuitive depiction of the analysis results. ClueGO is enhanced with CluePedia, which allows for a thorough examination of pathways (42). WikiPathways is a collaborative, open-source tool for curating biological pathways. Metascape selects the most informative phrases from the GO clusters obtained using a heuristic method. It takes a sample of the 20 highest-scoring clusters, picks up to 10 best-scoring words (lowest *p*-values) inside each cluster, and then connects all term pairs with Kappa similarity >0.3 (Figure 4). Cytoscape is used to show the resulting network, with each node representing one enriched phrase. The cluster IDs or the *p*-values can be used to color the nodes. Edges connect phrases that are similar—the thicker the edge, the greater the resemblance (43). To maintain readability, only one label (corresponding to one term) is displayed per cluster (Figure 4).

### Hamiltonian Energy


$$\begin{array}{l} HE=E_{c}-\;\gamma N_{c}, \end{array}$$

where *E*_*c*_ represents number of edges in a cluster, *N*_*c*_ for number of nodes, and the gamma constant γ = 0.8. The HE calculation for a network within the modules considers contributions from the organization of nodes and edges in a competitive manner, and this energy is used in organizing or re-organizing the network at various levels. This approach can also magnify the significant changes in the network organization when it goes down to various levels of organization, which capture the importance of hubs in the network and at the modular level. Therefore, HE formalism proves to be a useful technique for considering variations in the network organization (44). For hubs at each level of all potential modules in the network, the HE was determined (Figure 2). When the network's HE is plotted as a function of network modules, we discover that the energy distribution is highest in the native network and gradually decreases as the amount of organization grows. Because HE is based on node and edge competition for a set resolution parameter value (gamma symbol), a drop in HE reflects the dominance of interacting edges over the network size, implying rapid information processing (45).

### Most Influential, Potential, and Sovereignty of the Nodes in a Network

**Figure 5:**
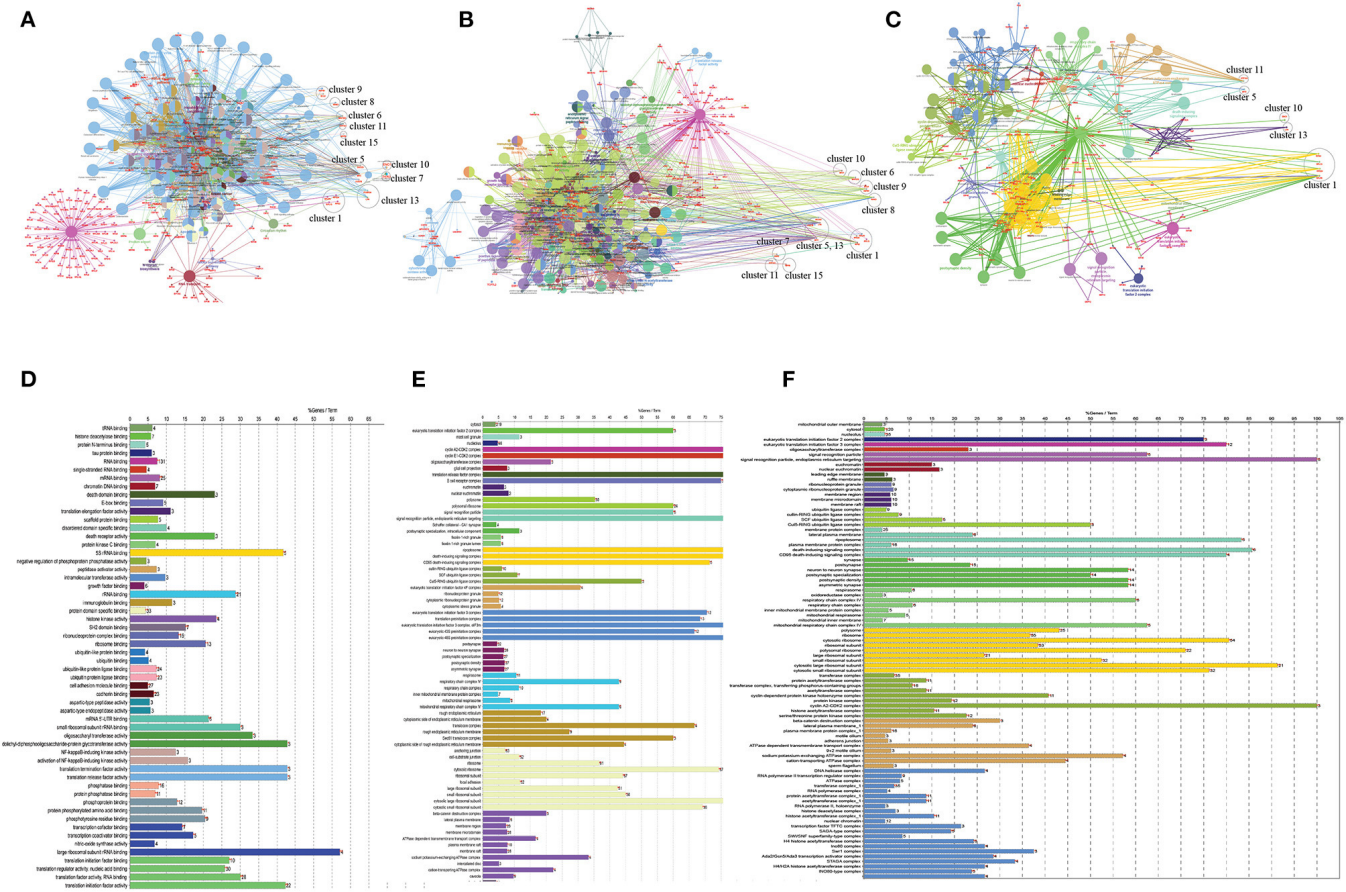
**(A)** Gene ontology study of hub genes (CVD and CKD) using ClueGO, a cytoscape plugin, including KEGG pathways **(B)**, molecular function **(C)**, and cellular localization, and **(D–F)** their respective bar plots. In the case of KEGG pathways, the network encompasses all modules and their seed genes. The modules are labeled with a circle and are referred to as clusters. There are four seed genes in the first cluster. The different colors of nodes indicate which genes are involved in which functions. All clusters are included in the molecular function, whereas only five clusters are visible in cellular localization (cluster 1, 5, 10, 11, and cluster 13).

Integrated Value of Influence (IVI) is a method for identifying the most influential nodes in a network that considers all topological aspects. The IVI formula combines the most important local (degree centrality and Cluster Rank), semi-local (neighborhood connectivity and local H-index), and global (betweenness centrality and collective influence) centrality measures in such a way that their effects are synergized and their biases are eliminated (46). The degree function from the igraph package can be used to calculate degree centrality, which is the most used local centrality measure. The degree centrality (DC) $DCi=\underset{i\;\neq \;j\;}{\sum }=A_{ij}$ is the simplest local centrality metric for a graph, where A represents the adjacency matrix of the associated network and Aij = 1 if nodes i and j are connected and Aij = 0, otherwise (47). Betweenness centrality, like degree centrality, is a widely used centrality metric. However, it only represents a node's global centrality. Another important centrality metric that reflects a node's semi-local centrality is neighborhood connectedness. For the first time, this centrality measure can be calculated in the R environment using the influential package. ClusterRank is another local centrality metric that removes the negative impacts of local clustering by intermediating between local and semi-local properties of a node. The H-index is a measure of how well a piece of the H-index is a semi-local centrality metric that was inspired by its use in analyzing the influence of researchers and is now available in the R environment for the first time via the influential package (48). Local H-index (LH-index) is a semi-local centrality measure and an upgraded variant of H-index centrality that applies the H-index to a node's second order neighbors and is now calculable in R using the influential package. The product of the reduced degree (degree – 1) of a node and the total reduced degree of all nodes at a distance d from the node is calculated as Collective Influence (CI). For the first time, this centrality metric is included in a R package. Two global centrality measures, betweenness centrality and collective influence, are among the most extensively used for identifying network influencers. Betweenness is the likelihood of a node in a network to be on the shortest path between nodes. Influencers of information flow inside a network are nodes with a high betweenness. Sometimes, we want to find the nodes that have the most potential for propagating information throughout the network, rather than the most influential nodes. ClusterRank, neighborhood connectedness, betweenness centrality, and collective impact all contribute to the spreading score, which is an integrative score made up of four separate centrality measurements. Also, one of the primary components of the IVI is the spreading score, which measures the spreading potential of each node within a network. In some circumstances, we wish to find out which nodes have the most sovereignty in their immediate surroundings. Hubness score is an integrative score comprised of two different centrality measures: degree centrality and local H-index (49). In addition, the Hubness score, which is one of the key components of the IVI, shows the power of each node in its surrounding environment (Figure 5).

## Discussion

The risk of CVD is increased with stages of CKD by promoting myocardial hypertrophy, coronary (CAD), atherosclerosis, and fluid overload. As the two most important organs of the body, the heart and kidneys work in close relation to each other. So, to unroll this methodical relationship at the molecular level, the present study was intervened. In this study, we have used integrated network-based approach and IVI methods to extracts the information (influential of nodes), hub genes, functions, and pathways. One of the limitations of this kind of study is that we have different cell and tissue types. Using p-adjustment is not the right choice under such circumstances as we did get no significant genes at 0.5 adjusted *P*-value for the GSE43484 and GSE67492. The current study is more like the meta-analysis of gene expression datasets using common microarray platforms which is quite widespread in many species. Apart from all the technicalities, the sharing of similar functions associated with transcripts of similar expression has persistently been used to annotate functions for humans in bioinformatics. Thus, using multiple datasets within functional premises governed by genes expressing in cell/tissue for a phenotype can be considered to involve similar pathways. This study retrieved 2,773 DEGs from 11 GSE Series (6 CVD and 5 CKD) series with thousands of genes. The 2,873 genes were overlapped in both cases during the process of distinguishing between the significant genes. Finally, based on our filtering criterion, we got to 43 key (or seed) genes in both conditions (24 down and 19 up-regulated genes) represented by Venn diagram in Figure 2.

Our retrieved key genes might be of significance in understanding the pathological degree of CRS in a better way. The network was constructed from 43 seed genes (487 nodes and 12,027 edges) using String (a web tools) with an interaction score of 0.9, the network was then imported to Cytoscape (3.8.1) for further downstream analysis. Out of 43, only 19 seed genes (8 up and 11 downregulated genes) were retained by the native network.

To find the involvement of the DEG(s) at different levels in the constructed network, the native or primary network was further broken down to 15 subnetworks or modules using MCODE (MCODE parameters were used for network scoring and cluster finding, i.e., “Degree cutoff = 2,” “node score cutoff = 0.2,” “k-score = 2,” and “max. depth = 100.” Out of these 15 modules, 10 modules were found to have seed genes. Interestingly, Module 1 contains 4 downregulated (RPL13, RPLP0, RPS2) and one upregulated (RPS24) gene, module 5 contains one downregulated gene (MYC) and two (SOCS3, COX7B) upregulated genes, module 6 contains two genes, one (MAP2K3) downregulated and one (TNFSF10) up-regulated gene. Modules 7 and 8 consist of one downregulated (PER3) and one (IRAK3) up-regulated gene. Module 9 contains two genes, one BCL6 (upregulated) and one HSPB1 (downregulated). Module 10 contains one downregulated gene (ENO1). Module 11 contains the gene ATP1A1 (downregulated), and module 13 and 15 contain two genes each, namely, LYN, FCGR3A (downregulated) and NPR3, NPPB (up-regulated) as shown in Table 3. As the number of nodes and edges decreases, the MCODE score decreases as shown in Figure 3. A high MCODE score indicates that the nodes are well-connected (dense network). To further establish the stability and integrity of these modules, Hamiltonian energy was calculated using the formalism in the method section where low HE suggests less likely to remain stable under stress (say when seed genes are removed). Module 7 shows very low value of HE, while modules 8 and 9 bar indicate higher values than module 5 (see Figure 3). The decline of top to bottom indicates the hierarchy of nodes. Seed genes (19) were further studied for their functions (GO TERM) and pathways. These 19 genes were submitted to Metascape, an online database, to get an understanding of the biological processes, molecular functions, and pathways they represent. Gene ontology terms are a comprehensible input of terms organized into three categories: Molecular function, Biological Processes, and Protein Class. Thus, these extracted DEGs were submitted to ClueGO, a Cytoscape plug-in that facilitates the biological interpretation, functional differences, and visualizes functionally grouped terms in the form of networks, tables, and charts (Figure 4). It illustrates the clusters of 8 networks based on the color of the gene connections and the percentage of genes in each cluster. The seed gene count, *p*-value, and gene symbol, along with their GO-TERM are represented in the table.

**Table 3:** The gene tracing in a network where the primary network is broken down into subnetworks/clusters/modules is shown in this table.

**Sr. No**.	**Module**	**Nodes**	**Edges**	**#Count**	**Gene of interest**
1	Module 1	133	8061	4	** RPL13, RPLP0, RPS2, RPS24 **
2	Module 5	44	231	3	** MYC, COX7B, SOCS3 **
3	Module 6	36	126	2	** MAP2K3, TNFSF10 **
4	Module 7	6	14	1	** PER3 **
5	Module 8	25	67	1	** IRAK3 **
6	Module 9	30	80	2	** BCL6, HSPB1 **
7	Module 10	16	38	1	** ENO1 **
8	Module 11	5	10	1	** ATP1A1 **
9	Module 13	5	6	2	** LYN, FCGR3A **
10	Module 15	3	3	2	** NPR3, NPPB **

*Ten modules out of fifteen have our gene of interest (seed genes). The table displays the number of nodes, edges, and genes in the specific module. Upregulation genes are shown in red, whereas downregulation genes are highlighted in green. There are a total of 19 genes that have been traced (11 downregulation and 8 up-regulation)*.

**Figure 6:**
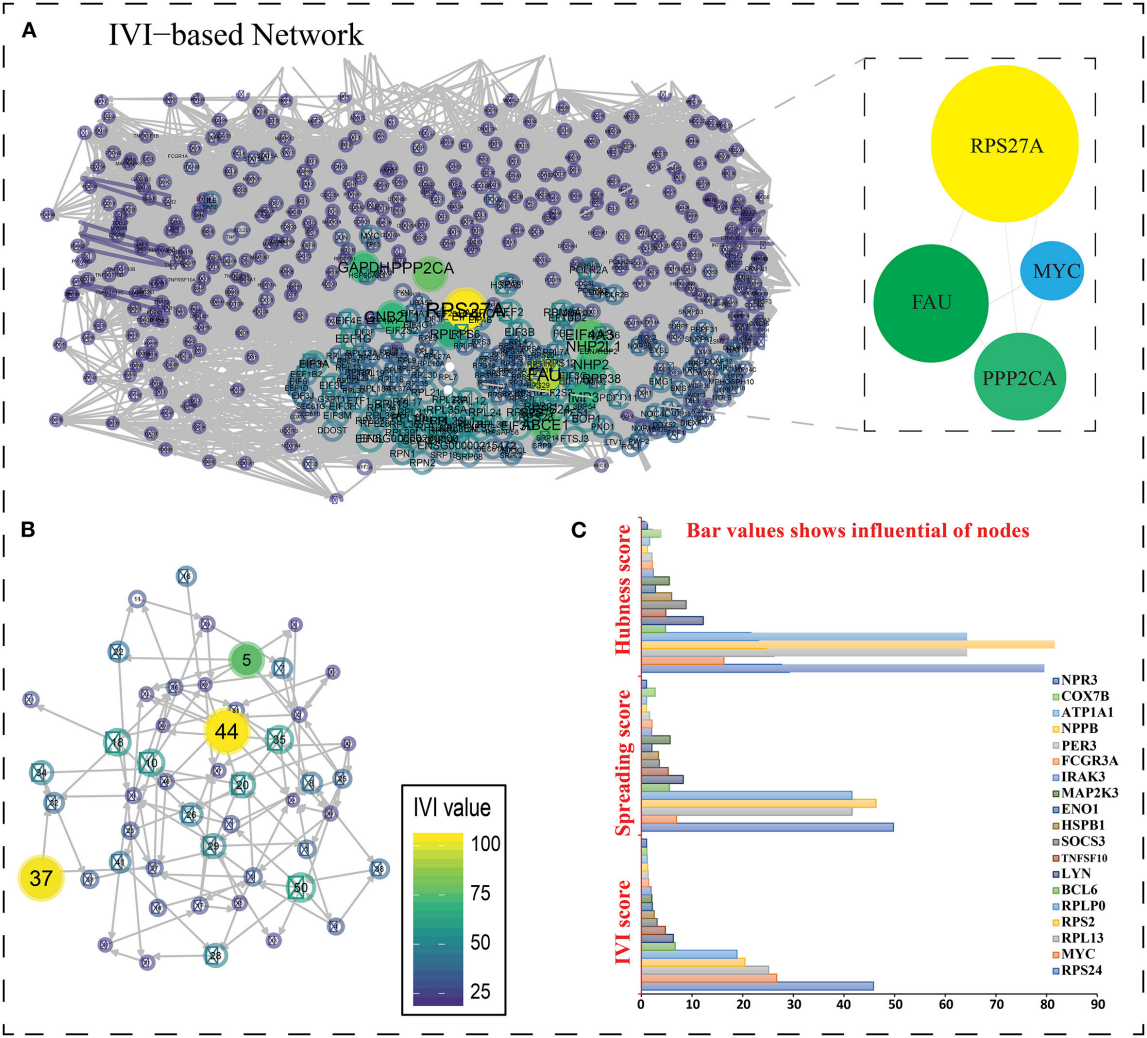
**(A)** The most important nodes are shown in the network created with R and influential packages. Zooming closer on the nodes reveals their color and interaction. The nodes in the native network are mostly the same color, indicating that they have a score of <25. The values of nodes are represented using a color spectrum. If the nodes are yellow, it suggests they are larger in size and have values >75. **(B)** Higher values are represented by a larger IVI value representation with yellow color. **(C)** A bar graph depicting three scores (IVI-Score, Spreading Score, and Hubness Score) for 19 essential genes' hub nodes. RPS27A is the highest non-seed gene in all three scores, with a value of 100, whereas RPS24 is the top seed gene in two of them (IVI-Score and Spreading score), with values of 45.82538 and 49.77075, respectively. In hubness score 81.57813, RPS2 is the top seed gene. The most powerful node in the network is RPS27A (non-seed gene).

**Figure 7:**
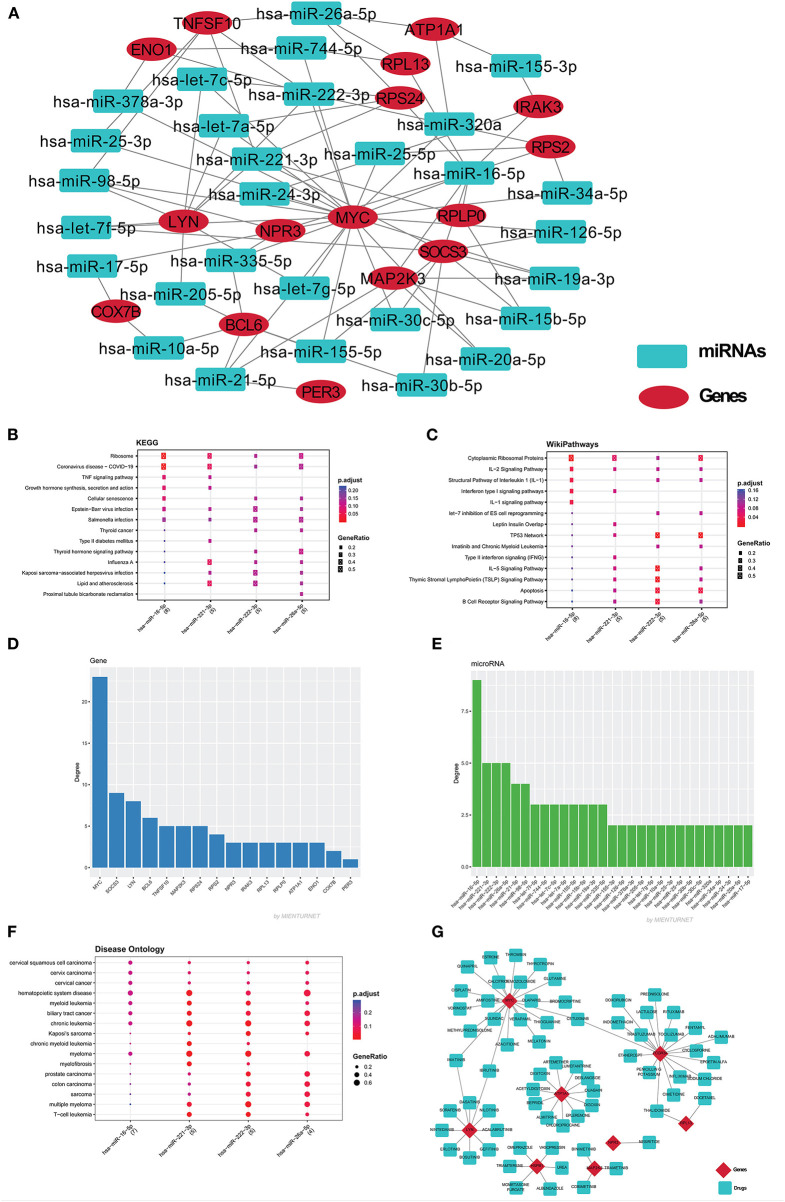
**(A)** The miRNAs-mRNA network contains 47 nodes and 85 edges. Red color eclipse indicates genes, whereas, cyan color rectangle indicates their associated miRNAs. The mienturnet database also analyzes the major miRNAs pathways of key miRNAs. The degree of these four miRNAs was plotted against the KEGG pathways **(B)**, WikiPathways **(C)**, and disease ontology **(F)**. The bar plot shows the genes **(D)** and miRNA **(E)** based on degree centrality. **(G)** The gene-drug network was build using drug gene interaction database, which build the network of 78 nodes and 72 edges. Red color diamond for genes and cyan color for their drugs.

Based on GO term enrichment, the highest represented a group of 8 genes (BCL6, ENO1, HSPB1, LYN, MYC, MAP2K3, SOCS3, and IRAK3) perform negative regulation of intracellular signal transduction (GO:1902532) while the least suggested function is negative regulation of apoptotic signaling pathway (GO:2001234) (see Figure 5A for seed gene pathways), with circles indicating modules. The different pathways are indicated by the color of interaction between nodes. If a gene has multiple colors, it suggests that it plays multiple roles in different pathways. Genes that play a key role in the pathophysiology of various diseases could be useful biomarkers in future research. In cluster 5, the gene MYC has many colors and various connections via edges. Thus, it may be a more important gene as compared to others. While some seed genes, like RPS24 and RPS2, show only one type of color that suggests a unique function that also plays a key role (see Figures 5B,C for molecular functions and cellular location of seed genes). Following the PPIN and Gene enrichment analyses, we tried to find the most influential nodes, also known as hub nodes, using the IVI-value and other associated scores. The IVI values, hubness score, spreading score, and local HI index of the 19 seed nodes are displayed in the network. The non-seed genes, such as RPS27A and FAU, have the greatest values. In Figure 6, the size of the nodes and colors indicates higher to lower IVI value. If the nodes have a higher value than 100, the color will be yellow and circle size enlarge. The exact IVI-value of nodes is mentioned in Table 4. The spreader nodes are projected to have the largest impact on the flow of information throughout the network because they have high connections with other nodes inside the network. We took these influential nodes to add one more intermediate layer of information in the networks that reveal the interaction of genes and proteins through the intermediate miRNA that regulate the genes (Figure 7). Thus, giving us a glimpse of the underlying physiology of these two conditions that were cumulatively called CRS.

**Table 4:** The top 20 nodes in the network were extract on the basis of IVI-score, spreading score, hubness score, CI score, and local HI score.

**Sr. No**.	**Gene**	**IVI-value**	**Gene**	**Spreading score**	**Gene**	**Hubness score**	**CI**	**Gene**	**Local HI**	**Gene**
1	RPS27A	100	RPS27A	100	RPS27A	100	1453138	EIF4G1	19288	RPS27A
2	FAU	86.54204	UBA52	66.35965	UBA52	83.6756	919132	FAU	17854	UBA52
3	PPP2CA	69.49985	EIF2S2	53.61083	RPS6	82.12102	910888	RPL10	17813	RPS6
4	EIF4A3	66.75017	EIF3M	53.49261	RPS2	81.57813	897390	RPL10A	17813	RPS2
5	ABCE1	65.97182	EIF3C	53.38627	RPS7	81.44018	866059	RPL10L	17792	RPS14
6	NHP2L1	64.97156	EIF3J	53.20541	RPS9	81.44018	862729	RPL11	17761	RPS7
7	GAPDH	61.76868	EIF2S1	53.19844	RPS14	81.30669	816804	RPL12	17692	RPS9
8	GNB2L1	60.52372	EIF3D	52.99297	RPS3	80.93769	806190	RPL13	17649	RPS3
9	IMP3	56.46971	EIF3K	52.99297	RPS18	80.63545	774473	RPL13A	17606	RPS11
10	NHP2	56.35967	EIF3B	52.53035	RPS11	80.50452	769952	RPL14	17606	RPS18
11	RPL11	55.67579	EIF5	52.38929	RPS27	80.35306	761514	RPL15	17606	RPS27
12	RPP38	51.82545	EIF3F	52.28384	RPS13	80.3332	751443	RPL17	17606	RPS13
13	EIF3D	46.9192	EIF3H	52.28384	RPS16	80.3332	681471	RPL18	17606	RPS15
14	RPS24	45.82538	EIF1AX	52.28095	RPS17	80.3332	674001	RPL18A	17606	RPS16
15	EEF1G	45.05756	PPP2CA	51.95736	RPS23	80.3332	646646	RPL19	17606	RPS17
16	BOP1	44.26816	RPS14	51.83609	RPS3A	80.3332	634176	RPL21	17606	RPS23
17	EIF3L	43.55821	RPS7	51.10068	RPS4X	80.3332	630175	RPL22	17598	RPS3A
18	RPL24	42.99506	RPS9	51.10068	RPS5	80.3332	624200	RPL22L1	17584	RPS4X
19	RPL14	42.20691	GNB2L1	50.96442	RPS8	80.3332	622336	RPL23	17548	RPS5
20	RPS28	41.40638	SEC61B	50.94699	RPS15	80.27672	621550	RPL23A	17548	RPS8

*These 20 nodes/genes are the most important nodes in the network retrieved from IVI methods. Using versatile methods (IVI-methods), most influential network nodes can be detected*.

**Figure 8:**
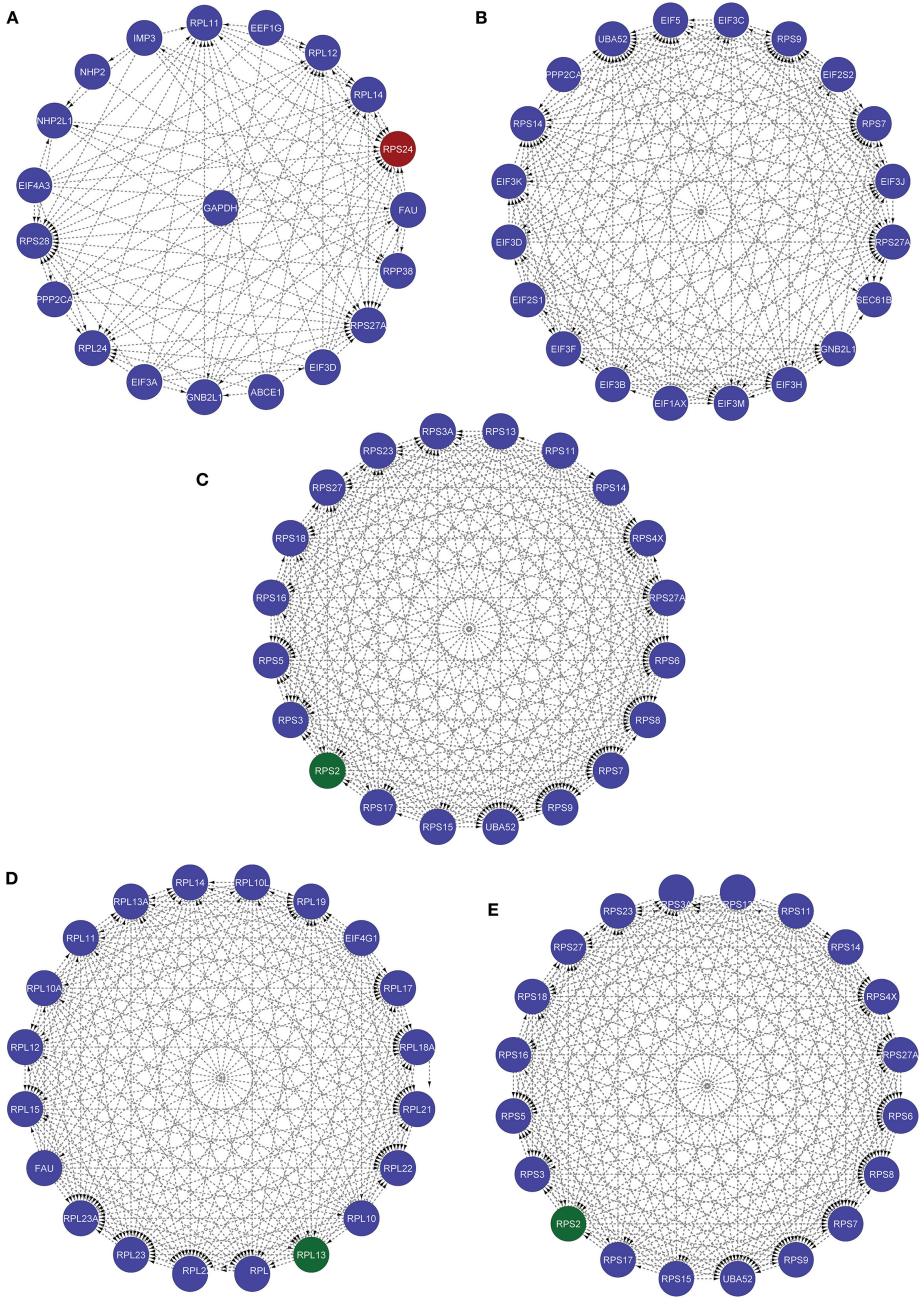
These five networks containing the top 20 nodes were extracted from the native network using five centrality approaches. **(A)** Based on IVI method, RPS24 seed genes is present. **(B)** According to the Spreading Score, there are no seed genes in the top 20 nodes. **(C)** Local H index network, **(D)** collective influence network, **(E)** hubness score network with RPS2 seed gene.

Furthermore, we used various other available tools for identifying the most influential nodes with IVI like based on its centrality measurements (9, 10). The DC is the simplest local centrality measure for a graph (degree centrality). Two global centrality measures, betweenness centrality, and collective influence, are among the most extensively used for identifying network influencers. Betweenness is the tendency of a node in a graph to be on the shortest path between nodes (1). Influencers of information flow inside a network are nodes with a high betweenness (11). The collective number of nodes that may be reached from a given node is measured by collective impact, a new global centrality metric. Neighborhood connectedness is a network's semi-local centrality measure that considers node connectivity (number of neighbors). It's called a semi-local measure because it's not limited to a node's immediate neighbors and considers the entire environment. The average connectivity of all neighbors of a vertex I is defined as its neighborhood connectivity. It is also said that a node's prominence in the network is determined by not only the number of first connections (degree centrality), but also the amount to which the nodes near neighbors are connected to each other and other nodes (neighborhood connectivity). The greatest value h such that there are at least h neighbors of a degree greater than or equal to h is defined as the H index of node i. The local H index is a semi-local centrality metric, despite its name, because it applies the H index centrality to a node's second order neighbors. All these centrality metrics (Hubness Score, Spreading Score, Degree Centrality, ClusterRank, local H index, neighborhood connectivity, betweenness centrality, and collective influence) are critical for identifying a network's most significant nodes (Table 5). Based on five centrality top 20 genes network extract from native network (Figure 8).

**Table 5:** The scores of the 19 seed genes in the native network were determined.

**Sr. No**.	**Gene**	**IVI score**	**Gene**	**SPD score**	**Gene**	**Hubness score**
1	RPS24	45.82538	RPS24	49.77075	RPS2	81.57813
2	MYC	26.69248	RPS2	46.32048	RPS24	79.5567
3	RPL13	25.10682	RPL13	41.60822	RPL13	64.30335
4	RPS2	20.43554	RPLP0	41.56268	RPLP0	64.30078
5	RPLP0	18.87599	LYN	8.263571	MYC	16.28468
6	BCL6	6.661571	MYC	6.925186	LYN	12.20791
7	LYN	6.297702	MAP2K3	5.662946	SOCS3	8.827513
8	TNFSF10	4.720607	BCL6	5.502842	HSPB1	5.966899
9	SOCS3	3.093086	TNFSF10	5.278298	MAP2K3	5.497646
10	HSPB1	2.515714	SOCS3	3.546049	TNFSF10	4.816811
11	ENO1	2.15667	HSPB1	3.370381	BCL6	4.770602
12	MAP2K3	2.067014	COX7B	2.702907	COX7B	3.981947
13	IRAK3	1.913351	FCGR3A	2.099974	ENO1	2.789033
14	FCGR3A	1.432519	ENO1	2.067738	IRAK3	2.3437
15	PER3	1.328185	IRAK3	2.014448	FCGR3A	2.182645
16	NPPB	1.24651	PER3	1.614294	PER3	2.082526
17	ATP1A1	1.15923	ATP1A1	1.031002	ATP1A1	1.611522
18	COX7B	1.088852	NPPB	1	NPPB	1.19186
19	NPR3	1.030828	NPR3	1	NPR3	1.19186

*RPS24 has the greatest score in the table, while NPR3 has the lowest*.

These results offer DEGs that may act as therapeutic targets for CVD and CKD in the future. This study reveals many aspects of CVD and CKD, such as gene-gene interaction, gene enrichment analysis, and pathways of hub genes. The hub genes may be the biomarker of the two conditions (CVD and CKD) need to be validated further but there are limitations such as few genes may be up-regulated and downregulated in the separate entity (means either in CVD or CKD). Maybe those genes are the key genes in CVD or CKD further research will reveal this gap. The study mainly focuses only on overlapped genes, despite all up and down genes. The non-seed genes are the most influential nodes in the network, not related to CVD and CKD. Because of the PPIN networks from the String database construct, the gene-gene interaction network using the source selected (human) and not disease specific genes interact with the seed genes. So, the study focuses only on the seed genes or related genes with the disease, but the results show the IVI -score, hubness score, and Spreading score, etc., of the non-seed genes (Table 4).

Our computational approach offers a comprehensive study, revealing the biomarkers of CVD and CKD using network approach and IVI methods (centrality measures), signifying the importance of nodes in the network, which help in Discover and understanding the various aspects of this disease. CKD Patients exhibit a pronounced risk for CVD events, utmost 50% of patients with CKD (stage 4 to 5) have CVD.

## Conclusion

Evidence-based approach has always been the center of clinical studies, while *in-silico* approaches focus to produce that potential evidence based on past knowledge, thus making this integrated process fast and efficient. On the other hand, there is great efforts are ongoing with the aim of reducing CVD residual risk by developing reliable prognostic and predictive biomarkers. Apart from many challenges, finding seed genes is one of the first challenges and we have proposed a very simple and efficient way to do that using set theory. The resulting 43 seed genes (that defines the molecular relationship between CVD and CKD) can then be used to construct the GGiN (gene-gene interaction network) to uncover the possible biological and functional meaning. On the other hand, we have used IVI method to calculate the influence of the nodes in the network, which further minimizes the gene list to a more realistic one (that can be tested *in-vivo* or *vitro*). Our study finds 19 genes out of many for being more prominent in the CRS (published data), whereas many other genes show a good expressions level. Although, RPS27A, a non-seed gene, was found to be the most influential node in the network followed by RPS2 and MYC. Based on these findings, new validation experiments can be constructed to further prove these as markers or good drug targets. Thus, giving us an opportunity to reduce the risks of CKD and CVD.

## Data Availability Statement

The datasets presented in this study can be found in online repositories. The names of the repository/repositories and accession number(s) can be found in the article/Supplementary Material.

## Author Contributions

MA and RI conceptualized the work. MA and AAla did data curation. MA, RA, SH, and AAls prepared the figures. MA, RA, ST, AAla, SA, and RI wrote the manuscript. HB, SA, MA, RI, and RB revised the manuscript. All authors contributed to the article and approved the submitted version.

## Funding

MA, ST, and RA were financially supported by the Indian Council of Medical Research, Government of India under Senior Research Fellow. FTS No. ISRM 11/(04)/2019. AAla financially was supported by the Department of Health and Research, (DHR) Ministry of Health and Family Welfare, Government of India under young scientist F.No. 12014/06/2019-HR.

## Conflict of Interest

The authors declare that the research was conducted in the absence of any commercial or financial relationships that could be construed as a potential conflict of interest. The reviewer MA declared a shared affiliation, with six of the authors MA, ST, RA, MS, AAla, and RI to the handling editor at the time of the review.

## Publisher's Note

All claims expressed in this article are solely those of the authors and do not necessarily represent those of their affiliated organizations, or those of the publisher, the editors and the reviewers. Any product that may be evaluated in this article, or claim that may be made by its manufacturer, is not guaranteed or endorsed by the publisher.

## Supplementary Material

Supplementary Table 1 In cardiovascular disease (CVD) and chronic kidney disease (CKD), total DEGs were gathered from 11 GSE series, as well as up and down common genes.

Supplementary Table 2 All genes rank order via Integrated Value of Influence (IVI) values.
